# Oleuropein-Induced Apoptosis Is Mediated by Mitochondrial Glyoxalase 2 in NSCLC A549 Cells: A Mechanistic Inside and a Possible Novel Nonenzymatic Role for an Ancient Enzyme

**DOI:** 10.1155/2019/8576961

**Published:** 2019-07-22

**Authors:** Cinzia Antognelli, Roberta Frosini, Maria F. Santolla, Matthew J. Peirce, Vincenzo N. Talesa

**Affiliations:** ^1^Department of Experimental Medicine, University of Perugia, Perugia 06129, Italy; ^2^Department of Pharmacy, Health and Nutritional Sciences, University of Calabria, 87036 Rende, Italy

## Abstract

Oleuropein (OP) is a bioactive compound derived from plants of the genus Oleaceae exhibiting antitumor properties in several human cancers, including non-small-cell lung cancer (NSCLC). Recent evidence suggests that OP has proapoptotic effects on NSCLC cells via the mitochondrial apoptotic pathway. However, the exact molecular mechanisms behind the apoptogenic action of OP in NSCLC are still largely unknown. Glyoxalase 2 (Glo2) is an ancient enzyme belonging to the glyoxalase system involved in the detoxification of glycolysis-derived methylglyoxal. However, emerging evidence suggests that Glo2 may have also nonenzymatic roles in some malignant cells. In the present study, we evaluated whether and how Glo2 participated in the proapoptotic effects of OP in NSCLC A549 cells. Our results indicate that OP is able to induce apoptosis in A549 cells through the upregulation of mitochondrial Glo2 (mGlo2), mediated by the superoxide anion and Akt signaling pathway. Moreover, our data shows that the proapoptotic role of mGlo2, observed following OP exposure, occurs via the interaction of mGlo2 with the proapoptotic Bax protein. Conversely, OP does not alter the behavior of nonmalignant human BEAS-2B cells or mGlo2 expression, thus suggesting a specific anticancer role for this bioactive compound in NSCLC. Our data identify a novel pathway through which OP exerts a proapoptotic effect in NSCLC and suggest, for the first time, a novel, nonenzymatic antiapoptotic role for this ancient enzyme in NSCLC.

## 1. Introduction

### 1.1. OP and Cancer

Oleuropein (OP) is an olive-derived polyphenol with an array of pharmacological properties, including anti-inflammatory and antioxidant effects [1], which have fostered intense interest in cancer research as a putative anticancer agent. Specifically, several lines of *in vitro* [2, 3] and *in vivo* [1] evidence demonstrate both antiproliferative and proapoptotic effects of this secoiridoid.

### 1.2. Lung Cancer and OP Effect on Non-Small-Cell Lung Cancer (NSCLC)

Lung cancer is the leading worldwide cause of cancer mortality [4]. While recent years have seen notable advances in the treatment of many cancers, the prognosis for non-small-cell lung cancer (NSCLC), which accounts for 85% of all lung cancer cases, remains dire. The lack of therapies capable of curing or even prolonging survival (estimated survival rate of only 15% at 5 years) of NSCLC highlights the urgent need for the identification of proteins and pathways responsible for the development and progression of the disease as potential drug targets for novel, effective, and safe therapeutic options. Epidemiologic studies have shown an inverse correlation between olive oil consumption and the risk of lung cancer [5–7], and recent findings have shown that OP exerts cytotoxic effects, by inducing mitochondrial apoptosis, in NSCLC cells [8, 9]. However, the molecular mechanisms driving the apoptogenic action of OP in NSCLC remain unclear.

### 1.3. Glyoxalases and Cancer

Glyoxalase 2 (Glo2) and glyoxalase 1 (Glo1) are together responsible for the detoxification of methylglyoxal (MG), a metabolic by-product of glycolysis with strongly proapoptotic effects [10]. Specifically, using glutathione (GSH) as a cofactor, Glo1 converts MG to S-D-lactoylglutathione (LSG) which is then hydrolyzed to D-lactate, regenerating GSH [10]. Recent evidence suggests that cancer cells show bioenergetic versatility depending on the genetic heterogeneity of the tumor [11, 12]. If on the one hand, many types of tumors consume larger amounts of glucose, compared to normal tissues, as Warburg originally observed, on the other hand, high glycolytic rates in tumors and mitochondrial respiration often operate simultaneously [11]. Moreover, it has been reported that cancer cells can also use a range of fuels including glutamine, fatty acids, heme flux, and acetate to sustain their growth and progression [11]. Cancers with enhanced glycolysis, such as prostate cancers [13, 14], are characterized by an increase in expression and enzyme activity of glyoxalases that reduce the production of MG, favoring escape from apoptosis [10, 15]. We have recently demonstrated, in prostate cancer cells, that Glo2 is involved in the control of apoptosis, in a Glo1-independent and possibly nonenzymatic manner, through the modulation of intracellular levels of p53 [16]. Here, we examined whether and how Glo2 might be involved in the proapoptotic effect of OP in NSCLC A549 cells and nonmalignant BEAS-2B cells.

## 2. Materials and Methods

### 2.1. Cells and Reagents

Human NSCLC A549 cells and human noncancerous BEAS-2B cells were purchased from Merck Spa (Milan, Italy). Cells were grown in RPMI-1640 (Thermo Fisher Scientific, Monza, Italy) supplemented with 10% heat-inactivated fetal bovine serum (FBS, Thermo Fisher Scientific, Monza, Italy) and 1% antibiotics (penicillin-streptomycin) (Thermo Fisher Scientific, Monza, Italy) at 37°C in a humidified incubator with 5% CO_2_. Confluent cells were treated with OP (Vinci-Biochem Srl, Florence, Italy) at final concentrations of 50 and 150 *μ*M (in dimethyl sulfoxide, DMSO) for 24 h [8, 9]. The most robust biological effects were obtained using 150 *μ*M OP 24 h post exposure, so mechanistic studies were carried out using this concentration. Untreated cells, incubated for the same time period, were used as controls. Separately, the effects of joint treatment with the specific Akt inhibitor MK2206 (10 *μ*M in DMSO for 48 hours) or SC79 Akt activator (4 *μ*g/ml in DMSO for 30 minutes) followed by 150 *μ*M OP for a further 24 hours were also examined. At the concentration, used MK2206 or SC79 exhibited no significant toxicity to cells. Unless otherwise stated, the biochemical evidence of inhibitor efficacy was established in preliminary experiments mainly by western blot analysis (data not shown). For agents in DMSO, final DMSO concentration in incubations was 0.01%. Controls contained an identical volume of DMSO vehicle. Laemmli buffer and the bicinchoninic acid (BCA) kit for protein quantification were from Thermo Fisher Scientific (Monza, Italy); Roti-Block was from Prodotti Gianni (Milan, Italy). The antibodies used in this study included the following: rabbit anti-Glo2 polyclonal antibody (pAb), mouse anti-Glo1 (D6) monoclonal antibody (mAb), mouse anti-rabbit anti-Bcl-XL pAb, rabbit anti-Bax (N20) pAb, rabbit anti caspase-3 pAb, and mouse anti-*β*-actin mAb from DBA Italia srl (Milan, Italy). Rabbit anti-p-Akt (Ser473) mAb was from Sigma-Aldrich (Milan, Italy); mouse anti-Bcl-2 mAb was from Dako (Milan, Italy); mouse anti-cytochrome c (Cyt c) mAb and mouse anti Apaf-1 mAb (clone 24) were from BD Pharmingen (Milan, Italy); mouse anti-Cyt c oxidase subunit IV (Cox IV) mAb was from Molecular Probes (Monza, Italy); mouse anti-SOD2 mAb was from Abcam (Milan, Italy).

### 2.2. Apoptosis Detection

Apoptosis was quantified by two methods, firstly by measuring the activation of caspase-3, using an enzyme-linked immunosorbent assay (ELISA) (Thermo Fisher Scientific, Monza, Italy), specific for activated human caspase-3 following the manufacturer's instructions, and secondly by DNA fragmentation using agarose gel electrophoresis as previously described [17, 18].

### 2.3. Enzymatic Activity Assays

Enzymatic activity assays were conducted on cell extracts prepared as previously described [19]. Briefly, cells treated (24 h) with OP were harvested and resuspended (10^7^ cells/ml) in 10 mM phosphate buffer pH 7.0, containing 1 mM dithiothreitol (for the detection of Glo2 and Glo1 enzymatic activities) and 0.1 mM phenylmethanesulphonylfluoride (PMSF). Cells were then homogenized with a Potter-Elvehjem homogenizer, and cell debris removed by centrifugation (13,000 x g for 30 min) and the resulting cell supernatants were assayed for protein content and enzymatic activity. Mitochondrial extracts were prepared using a Mitochondria/Cytosol Fractionation Kit (BioVision, Florence, Italy), according to the manufacturer's instructions. Protein concentration was determined with a bicinchoninic acid (BCA) kit (Pierce), by reference to a standard curve prepared with bovine serum albumin. Glo1 activity was assayed according to Mannervik et al. [20]. The assay solution contained 0.1 M sodium-phosphate buffer pH 7.2, 2 mM MG, and 1 mM GSH. Activity was measured spectrophotometrically by monitoring the increase of absorbance at 240 nm at 25°C. One unit of activity was defined as 1 *μ*mol of S-D-lactoylglutathione produced min^−1^. Conversely, Glo2 activity was assayed spectrophotometrically, at 25°C by recording the decrease in absorbance at 240 nm due to S-D-lactoylglutathione (0.3 mM) hydrolysis [21, 22]. One unit activity was defined as 1 *μ*mol S-D-lactoylglutathione hydrolyzed/min. Finally, SOD activity was measured using Calbiochem's Superoxide Dismutase Assay Kit II (EMD Chemicals, Gibbstown, NJ) according to the manufacturer's directions. One unit of SOD activity was defined as the amount of enzyme needed to exhibit 50% dismutation of the superoxide radical.

### 2.4. Cell Lysate Preparation for Western Blot

Cells (10^6^) were lysed in precooled radioimmunoprecipitation assay (RIPA) lysis buffer, proteins separated by SDS-PAGE, and subjected to Western blot analysis as previously described [23, 24]. Briefly, samples of equal protein concentration were mixed with Laemmli buffer and boiled for 5 minutes then resolved on 4-15% SDS-PAGE and blotted onto a nitrocellulose membrane (iBlot Dry Blotting System, Thermo Fisher Scientific, Monza, Italy). Membranes were blocked in Roti-Block for 1 h at room temperature, incubated overnight at 4°C with an appropriate dilution of the primary Abs. After washing with TBST, membranes were incubated (1 h, RT) with the appropriate HRP-conjugated secondary Ab and visualized using ECL (Amersham Pharmacia, Milan, Italy). The primary Ab was then stripped by incubating membranes in stripping buffer (100 mM 2-ME, 2% SDS, and 62.5 mM Tris-HCl, pH 6.8) and reprobed with an Ab against an appropriate housekeeping protein as an internal loading control.

### 2.5. Superoxide Anion Detection

Intracellular O_2_^·-^ production after OP treatment was detected using dihydroethidium (DHE) (Sigma-Aldrich, Milan, Italy) [25, 26]. DHE enters cells and reacts with the superoxide anion to form ethidium, which exhibits red fluorescence. Briefly, confluent A549 cells were exposed to OP and then incubated with 5 *μ*M DHE in HBSS (2 mM CaCl_2_, 1 mM MgSO_4_) at 37°C for 30 min. At the end of the incubation, cells were detached by scraping and disrupted by sonication. After clarification (13000 x g, 5 min, 4°C), supernatants were collected and fluorescence was measured using a fluorimeter (Kontrol Instrument, SFM 25, Eching, Germany) (excitation 488 nm, emission wavelength 512 nm). The results were confirmed using an HE-based HPLC assay (data not shown).

### 2.6. Gene Silencing

Pools of four small interfering RNA (siRNA) oligonucleotides targeting SOD2 (siSOD2) (ON-TARGET plus SMART pool siRNA) or non-targeting siRNA oligonucleotides (siCtr) (ONTARGET plus siCONTROL) as a negative control (all from Dharmacon RNA Technologies, Carlo Erba, Milan, Italy) were transiently transfected into NSCLC A549 cells using DharmaFECT 1 transfection reagent (Dharmacon RNA Technologies, Carlo Erba, Milan, Italy), according to the manufacturer's instructions. Potential effects due solely to the transfection reagent were controlled by performing mock transfections without any siRNA (data not shown). Since the biological readouts examined here were indistinguishable in nontransfected, mock-treated, or siCtr-treated cells, the observed changes resulting from siSOD2 treatment were reported relative to siCtr-exposed cells only.

### 2.7. Immunoprecipitation

Immunoprecipitation (IP) was performed using Dynabeads Protein G Immunoprecipitation Kit (Thermo Fisher Scientific, Monza, Italy) according to the manufacturer's instructions. Briefly, supernatants of cells lysed in ice-cold RIPA lysis buffer containing a protease inhibitor cocktail were incubated (4 h, 4°C) with protein G Dynabeads to which a rabbit anti-Glo2 Ab had been prebound (O/N, 4°C). Immune complexes were recovered magnetically and analyzed by Western blot using either anti-Bax or anti-Glo2 antibodies as described above.

### 2.8. Statistical Analysis

All data were generated from three independent biological replicates and expressed as the means ± standard deviation (SD). One-way analysis of variance with Dunnett's correction was used to assess differences among groups when appropriate. The statistical significance, determined by Student's *t*-test, was set at *p* < 0.05.

## 3. Results and Discussion

### 3.1. The Proapoptotic Effect of OP Is Associated with Mitochondrial Glo2-Increased Expression in NSCLC A549 Cells

**Figure 1:**
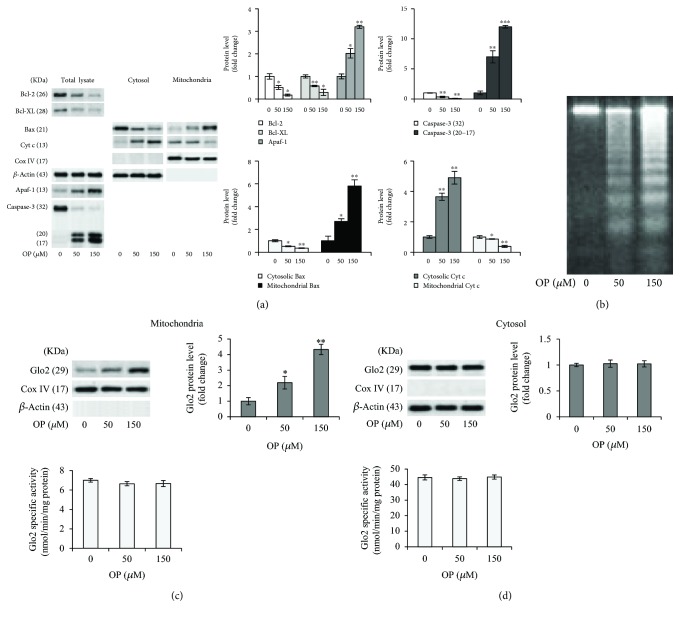
The proapoptotic effect of OP is associated with increased expression of mitochondrial Glo2 in NSCLC A549 cells. (a) Antiapoptotic Bcl-2 and Bcl-XL, proapoptotic Bax, cytochrome c (Cyt c), Apaf-1, and caspase-3 (intact protein, 32 kDa molecular weight; active fragments, 20 and 17 kDa molecular weight) protein expression in untreated (0 *μ*M) and oleuropein- (OP-) treated (50 and 150 *μ*M) A549 cells. (b) Apoptosis was confirmed at morphological level by DNA fragmentation, evaluated by gel electrophoresis. Electrophoresis is a representative of three independent experiments providing the same result. Evaluation of Glo2 expression, by western blot, and enzyme specific activity, by a spectrophotometric assay, in the (c) mitochondria and (d) cytosolic lysates of A549 cells. Histograms indicate the means ± SD of three different cultures, each of which was tested in quadruplicate and expressed as a percentage of control. Western blot analysis of *β*-actin or CoxIV expression is provided to show equal loading of the samples and to demonstrate successful enrichment of mitochondria in fractionated extracts. Blots are representative of three independent experiments, which gave the same results. ^∗^*p* < 0.05, ^∗∗^*p* < 0.01, and ^∗∗∗^*p* < 0.001, significantly different from control untreated cells.

The proapoptotic effect of OP in NSCLC A549 cells and the related intrinsic apoptosis mechanism were evaluated by measuring the levels of major proteins typically activated in a mitochondrial apoptotic pathway, namely, the antiapoptotic Bcl-2 or Bcl-XL or the proapoptotic Bax proteins, Cyt c, Apaf-1, and the final executioner caspase-3 [18] by immunoblotting. As shown in Figure 1(a), we found a significant dose-dependent decrease in the levels of the antiapoptotic Bcl-2 or Bcl-XL proteins paralleled by a marked increase in the levels of the proapoptotic Bax protein as well as its translocation from the cytosol to the mitochondria, in OP-exposed cells compared to controls. Concomitantly, Cyt c release into the cytosol, as well as the activation of Apaf-1 and caspase-3, was observed (Figure 1(a)). DNA fragmentation into oligonucleosomes, a hallmark of apoptosis, confirmed the apoptotic responses at the morphological level, as evidenced by the typical DNA laddering response (Figure 1(b)). Hence, in line with the literature [8, 9], our results show that OP induces apoptosis in NSCLC A549 cells through a mitochondrial pathway. Glo2 is an ancient enzyme that together with Glo1 participates in the removal of cytotoxic MG [10]. Very little is known about Glo2, including its role in MG scavenging and its functional significance in health and disease [10, 15, 16]. We have recently demonstrated, in prostate cancer cells, that Glo2 is involved in the control of apoptosis, in a Glo1-independent and possibly nonenzymatic manner, through the modulation of intracellular levels of p53 [16]. In particular, Glo2 protected cancer cells from apoptosis [16]. In humans, two Glo2 isoforms have been identified, one in the cytosol (cGlo2) and one in the mitochondrion (mGlo2) [27]. These isoforms are encoded by a single Glo2 gene through alternate translational start sites [28]. In order to investigate whether Glo2 could be involved in OP-induced apoptosis, we studied the protein expression and specific activity of the Glo2 enzyme in the mitochondrial and cytosolic fractions of A549 cells exposed to OP. Unexpectedly, we found that OP induced a dose-dependent and statistically significant increase in mGlo2 protein levels without affecting the enzyme's specific activity (Figure 1(c)). Similarly, OP did not modify cGlo2 expression either at protein or at functional level (Figure 1(d)). Overall, these results indicated that the proapoptotic effect of OP is associated with an increase in mGlo2, suggesting a novel mechanism by which this natural bioactive compound exerts its apoptogenic function in NSCLC A549. Moreover, our findings suggest a proapoptotic role of Glo2, at least of the mitochondrial isoform and at least following OP exposure in NSCLC A549 cells. This role turns out to be opposite to that observed for Glo2 in prostate cancer cells, where conversely, an antiapoptotic nonenzymatic role of this protein was described [10]. Even though the antiapoptotic action of Glo2 in prostate cancer cells has been shown without discriminating between the mitochondrial or cytosolic isoform, our findings highlight a potential and intriguing complexity of Glo2 biology, providing powerful motivation for further research. Moreover, these data showed that mGlo2 involvement in the apoptosis driven by OP is independent from its traditional function as a metabolic enzyme, positing this isoform as a “moonlighting” protein (a protein with more than one function), as has been documented for other “ancient” metabolic enzymes [29, 30], and in agreement with the emerging role for Glo2 in other malignant cells [16].

### 3.2. OP Induces Apoptosis through a Mechanism Involving SOD2-Mediated Superoxide Anion-Dependent mGlo2 Upregulation in NSCLC A549 Cells

**Figure 2:**
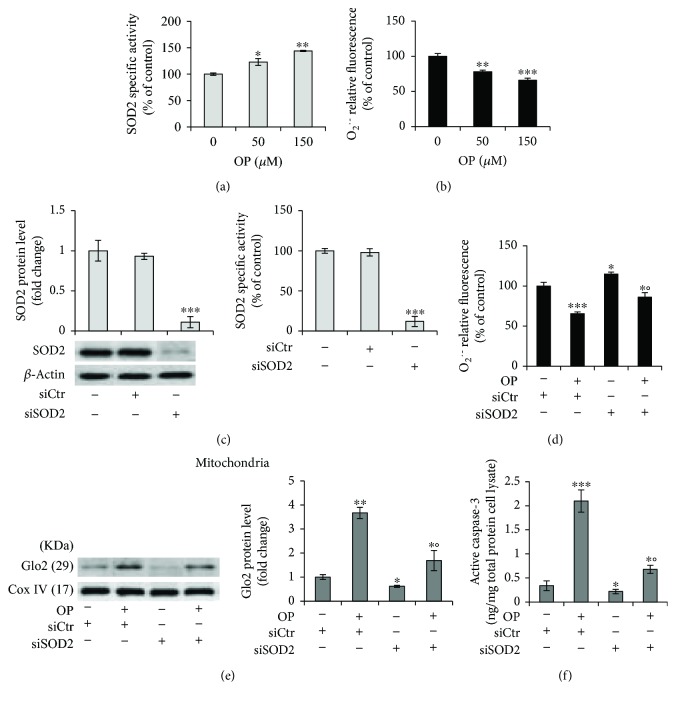
OP induces apoptosis through a mechanism involving SOD2-mediated superoxide anion- (O_2_^·-^-) dependent mGlo2 upregulation in NSCLC A549 cells. (a) Superoxide dismutase 2 (SOD2) activity and (b) superoxide anion (O_2_^·-^) level in untreated (0 *μ*M) and oleuropein- (OP-) treated A549 cells. Under 150 *μ*M OP exposure, SOD2 silencing by small interfering RNA (siSOD2) (c) significantly reversed (d) O_2_^·-^ levels, (e) mitochondrial Glo2 expression, and (f) apoptosis, evaluated by active caspase-3 expression. Western blots were stripped of the bound Abs and reprobed with anti-*β*-actin or anti-Cox-IV, to confirm equal loading. The western blots shown are representative of three separate experiments. Histograms indicate the means ± SD of three different cultures each of which was tested in triplicate. siCtr: control (nonspecific siRNA), (-) untreated, and (+) treated cells; ^∗^*p* < 0.05, ^∗∗^*p* < 0.01, and ^∗∗∗^*p* < 0.001 versus unexposed cells; °*p* < 0.05 versus OP-treated cells.

It has been shown that OP exerts antioxidant effects either directly by reducing the generation of reactive oxygen species (ROS) or indirectly through modulating endogenous antioxidant enzymes [31, 32]. In particular, it has been reported that OP has a potent superoxide anion scavenging activity [32]. In addition, because the mitochondria are the major sources of ROS production, mGlo2 may be somehow affected by the redox status of these organelles. Finally, it is known that Mn-superoxide dismutase (SOD2) plays a crucial role in the control of the mitochondrial apoptotic pathway [33]. Hence, we investigated whether OP-induced apoptosis occurred through the SOD2-mediated depletion of the superoxide anion (O_2_^·-^). As shown in Figure 2, OP induced a significant increase in SOD2 activity compared with untreated cells (Figure 2(a)) and this was paralleled by a marked decrease in O_2_^·-^ levels (Figure 2(b)). More importantly, striking SOD2 silencing, demonstrable by measurements of both protein expression and levels of enzyme activity (Figure 2(c)), was able, following 150 *μ*M OP exposure, to restore normal O_2_^·-^ levels (Figure 2(d)) and to decrease mGlo2 expression (Figure 2(e)) and apoptosis (Figure 2(f)).

Collectively, our results define a novel mechanism, based on the involvement of SOD2, O_2_^·-^, and mGlo2 in the proapoptotic effect of OP, thus adding further insight into the molecules activated by this bioactive compound in inducing apoptosis. The issue of whether the action of OP on malignant cells is predominantly antioxidant or prooxidant remains to be established. While in many cases, the proapoptotic effects of OP on tumor cells are elicited via pathways involving ROS generation and oxidative stress [34, 35], in others, OP has been reported to mediate antioxidant effects. Our results are in agreement with the studies supporting this last assessment [36, 37]. Moreover, our data suggest a role for mGlo2 in the group of the proteins participating in ROS-mediated apoptosis, thus providing further insight to the mechanisms underlying ROS-dependent apoptosis, which are still far from being completely understood [38].

### 3.3. OP Drives Apoptosis in NSCLC A549 Cells by Promoting mGlo2 Association to Bax

**Figure 3:**
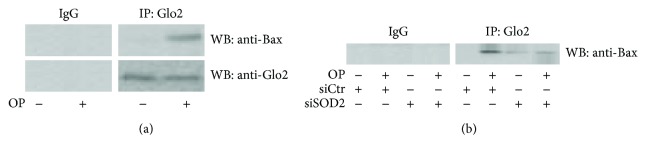
OP drives apoptosis in NSCLC A549 cells by promoting mGlo2 association to Bax. (a) Mitochondrial lysates from A549 cells treated with 150 *μ*M oleuropein (OP) were immunoprecipitated with protein G agarose-coupled anti-Glo2 (IP : Glo2) and subjected to western blotting (WB) with the anti-Bax antibody (WB : anti-Bax). Blots were then stripped and reprobed with the anti-Glo2 antibody to ensure equal immunoprecipitation of Glo2 protein. Mouse IgG was used as a negative control for immunoprecipitation; (b) mitochondrial lysates from A549 cells treated with 150 *μ*M OP under SOD2 silencing (siSOD2) or control (siCtr, nonspecific siRNA) were immunoprecipitated with protein G agarose-coupled anti-Glo2 (IP : Glo2) and subjected to western blotting (WB) with the anti-Bax antibody (WB : anti-Bax).

To begin to address the mechanism by which mGlo2 could promote apoptosis after OP exposure, we decided to address whether mGlo2 could interact with proteins involved in the known mitochondrial apoptosis pathway. We found that OP promoted mGlo2 association with the proapoptotic Bax protein (Figure 3(a)) and this was partially reversed by SOD2 silencing (Figure 3(b)).

Thus, our data provide evidence of a proapoptotic role for mGlo2 after OP exposure as well as a physical association with a known mediator of apoptosis, Bax. It has been demonstrated that during apoptosis, Bax and Bak mediate the release of cytochrome c from the mitochondria by clustering on the outer mitochondrial membrane and thereby increasing its permeability [39]. However, it remains unclear how outer membrane openings form. Based on our data, we hypothesize that Glo2 might help in achieving this goal, a possibility which needs further investigation. Our data on the interaction between Glo2 and Bax, although novel, are in fact in agreement with the proposal that Glo2 might form specific protein-protein interactions with its enzyme substrates as reported by Ercolani et al. [40]. Glyoxalases are ubiquitous enzymes. In yeast, it has been reported that the mGlo2 complements the cytosolic form in the detoxification of MG [28]. The role of mGlo2 in humans has been less investigated, and in agreement with our results, it does not appear to have an MG-scavenging function [16]. Recently, Navarro et al. have described an antiglycative role of OP in the HepG2 cell line [41]. In particular, they found that OP was able to trap MG, the cytotoxic metabolite preferentially detoxified by cGlo2 in cooperation with Glo1. We speculate that cGlo2 might not participate in the apoptotic effects of OP since OP directly reduces levels of MG.

### 3.4. OP-Induced mGlo2 Upregulation Is Dependent on p38 MAPK and Akt Signaling Pathways

**Figure 4:**
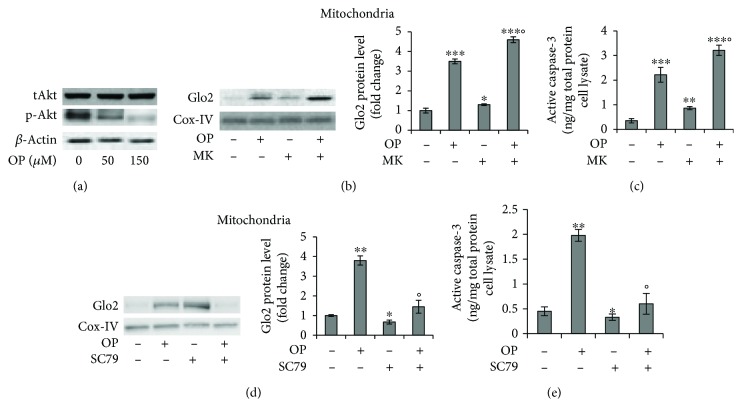
OP-induced mGlo2 upregulation is dependent on p38 MAPK and Akt signaling pathways. (a) Protein level of total Akt (tAkt) phospho-Akt (p-Akt) by western blot (WB) analysis after oleuropein (OP) treatment for 24 hours, (b, d) mitochondrial Glo2 expression, and (c, e) apoptosis, evaluated by WB and active caspase-3 expression, respectively, in A549 cells pretreated with the selective Akt inhibitor MK2206 (MK) (b, c) or the Akt activator SC79 (d, e) and following OP exposure (150 *μ*M). Western blots were stripped of the bound Abs and reprobed with anti-*β*-actin or Cox-IV, to confirm equal loading. The western blots shown are the representative of three separate experiments. Histograms indicate the means ± SD of three different cultures each of which was tested in triplicate. (-) untreated and (+) treated cells; ^∗^*p* < 0.05, ^∗∗^*p* < 0.01, and ^∗∗∗^*p* < 0.001 versus unexposed cells; °*p* < 0.05 versus OP-treated cells.

One of the most frequent events in carcinogenesis is the hyperactivation of the Akt signaling pathway [42, 43]. In NSCLC, activation of the Akt pathway promotes tumor progression by inducing evasion of apoptosis [44]. Here, we wanted to investigate whether the OP-induced increase in mGlo2 expression was paralleled by Akt desensitization and apoptosis induction. We first showed that Akt signaling is active in basal A549 cells and that OP reduced its activation (Figure 4(a)). Subsequently, by using the selective MK2206 (MK) Akt inhibitor, we demonstrated that OP-induced mGlo2 expression was upregulated by Akt deactivation and this was associated with a reversal of apoptosis. In fact, following OP administration, MK treatment potentiated mGlo2 protein expression (Figure 4(b)) and apoptosis (Figure 4(c)). Akt activation by SC79 [45] further confirmed that OP-induced mGlo2 upregulation is Akt-dependent (Figures 4(d) and 4(e)). The control of mGlo2 expression by Akt deactivation in our model is further supported by our observation that O_2_^·-^ depletion mediated an additional increase in the mGlo2 protein level after OP exposure. In fact, it was previously reported that O_2_^·-^ sustains A549 cell survival by supporting Akt activation [46]. Hence, it is plausible to assume that OP-induced depletion of O_2_^·-^ in our model leads to Akt deactivation and, in turn, to the upregulation of mGlo2.

### 3.5. OP Effect on the Viability and Glyoxalase Expression in Nonmalignant BEAS-2B Cells

**Figure 5:**
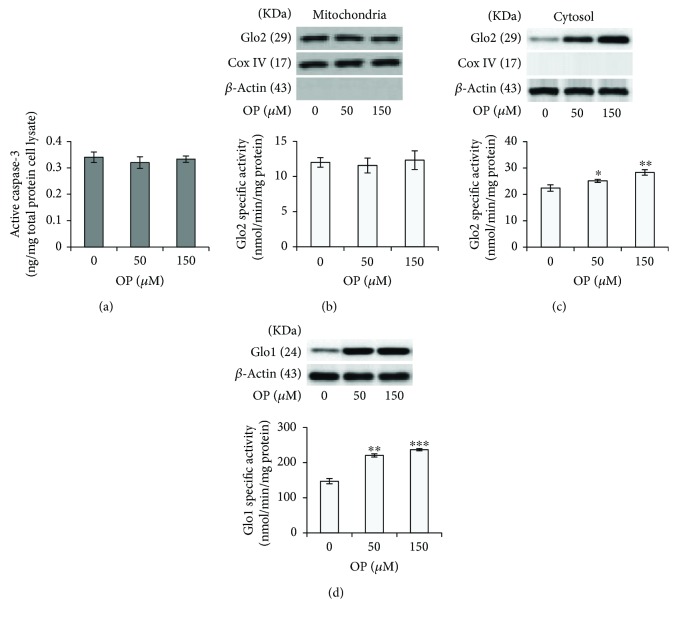
OP effect on the viability and Glyoxalase expression in nonmalignant BEAS-2B cells. (a) Apoptosis, evaluated by detecting active caspase-3 (b) mitochondrial and (c) cytosolic Glo2 expression and enzyme activity. (d) Glo1 protein levels and activity in A549 cells treated with OP. Protein expression and enzyme activities were evaluated by western blotting (WB) and specific spectrophotometric methods, respectively. Western blots were stripped of the bound Abs and reprobed with anti-*β*-actin or Cox-IV, to confirm equal loading. The western blots shown are the representative of three separate experiments. Histograms indicate the means ± SD of three different cultures each of one was tested in triplicate. ^∗^*p* < 0.05, ^∗∗^*p* < 0.01, and ^∗∗∗^*p* < 0.001 versus unexposed cells.

Most conventional anticancer therapies do not categorize between cancerous and normal cells, leading to unwanted side effects and toxicity. In agreement with the literature [8], we found here that OP did not affect the viability of normal BEAS-2B cells (data not shown). Moreover, no apoptosis was observed after OP exposure (Figure 5(a)), thus suggesting a selective toxicity against A549 cancer cells while sparing healthy, nonmalignant cells. Moreover, in BEAS-2B cells, OP did not affect mGlo2 expression or enzyme activity (Figure 5(b)), thus suggesting that the viability of BEAS-2B cells and malignant A549 cells is regulated by different proteins and mechanisms. Intriguingly, OP increased the protein expression and enzyme activity of both the cytosolic Glo2 isoform (Figure 5(c)) and Glo1 (Figure 5(d)), which needs further investigation.

## 4. Conclusions

**Figure 6:**
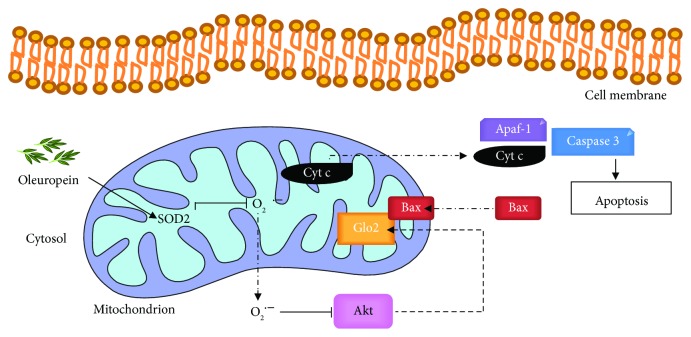
Oleuropein induces apoptosis in NSCLC A549 cells through mitochondrial glyoxalase 2 (Glo2). Oleuropein induces (→) SOD2 upregulation and consequent scavenging (**⊣**) of superoxide anion O_2_^·-^. The depletion of O_2_^·-^ inhibits (**⊣**) the Akt signaling pathway that, in turn, induces (→) the upregulation of mitochondrial Glo2 expression. Glo2 interacts with the proapoptotic protein Bax activating apoptosis through the intrinsic pathway [cytochrome c (Cyt c) release from the mitochondrion, activation of Apaf-1, and eventually caspase-3 activation].

The data reported here show that OP induces apoptosis in NSCLC A549 cells through a novel mechanism involving the SOD2/O_2_^·-^/Akt/mGlo2 axis (Figure 6), thus identifying mGlo2 as a crucial protein in OP-driven apoptosis and extending the limited information available on the anticancer effect of OP in NSCLC models [8, 9]. In addition, our results further supporting the idea that the pro-apoptotic role of OP in NSCLC cells, together with the absence of toxic effects on healthy cells, make this bioactive natural compound an excellent candidate for treating this malignancy.

## Data Availability

The data used to support the findings of this study are available from the corresponding author upon request.
