# Corrigendum to “Oleuropein-Induced Apoptosis Is Mediated by Mitochondrial Glyoxalase 2 in NSCLC A549 Cells: A Mechanistic Inside and a Possible Novel Nonenzymatic Role for an Ancient Enzyme”

**DOI:** 10.1155/2020/3045908

**Published:** 2020-09-09

**Authors:** Cinzia Antognelli, Roberta Frosini, Maria F. Santolla, Matthew J. Peirce, Vincenzo N. Talesa

**Affiliations:** ^1^Department of Experimental Medicine, University of Perugia, Perugia 06129, Italy; ^2^Department of Pharmacy, Health and Nutritional Sciences, University of Calabria, 87036 Rende, Italy

In the article titled “Oleuropein-Induced Apoptosis Is Mediated by Mitochondrial Glyoxalase 2 in NSCLC A549 Cells: A Mechanistic Inside and a Possible Novel Nonenzymatic Role for an Ancient Enzyme” [1], there were apparent discontinuities in the western blots in Figures 2 and 4, as raised in PubPeer [2]. The authors provided an explanation, the original files used to prepare the figures, and independent replicates, which are available in Supplementary Materials.

## Supplementary Materials

Supplementary Materials The authors provided a “letter of clarification,” the original files for Figures 2 and 4, and independent replicates.
