# Supplementary material for: Corrigendum to “Oleuropein-Induced Apoptosis Is Mediated by Mitochondrial Glyoxalase 2 in NSCLC A549 Cells: A Mechanistic Inside and a Possible Novel Nonenzymatic Role for an Ancient Enzyme”
Source: Oxid Med Cell Longev. 2020 Sep 9;2020:3045908. doi: 10.1155/2020/3045908 (PMC7502119; doi:10.1155/2020/3045908)
Supplement: Supplementary Materials — The authors provided a “letter of clarification,” the original files for Figures 2 and 4, and independent replicates. [file 3045908.f1.zip › Independent experiments Fig. 2 (1).pptx]

## Slide 1
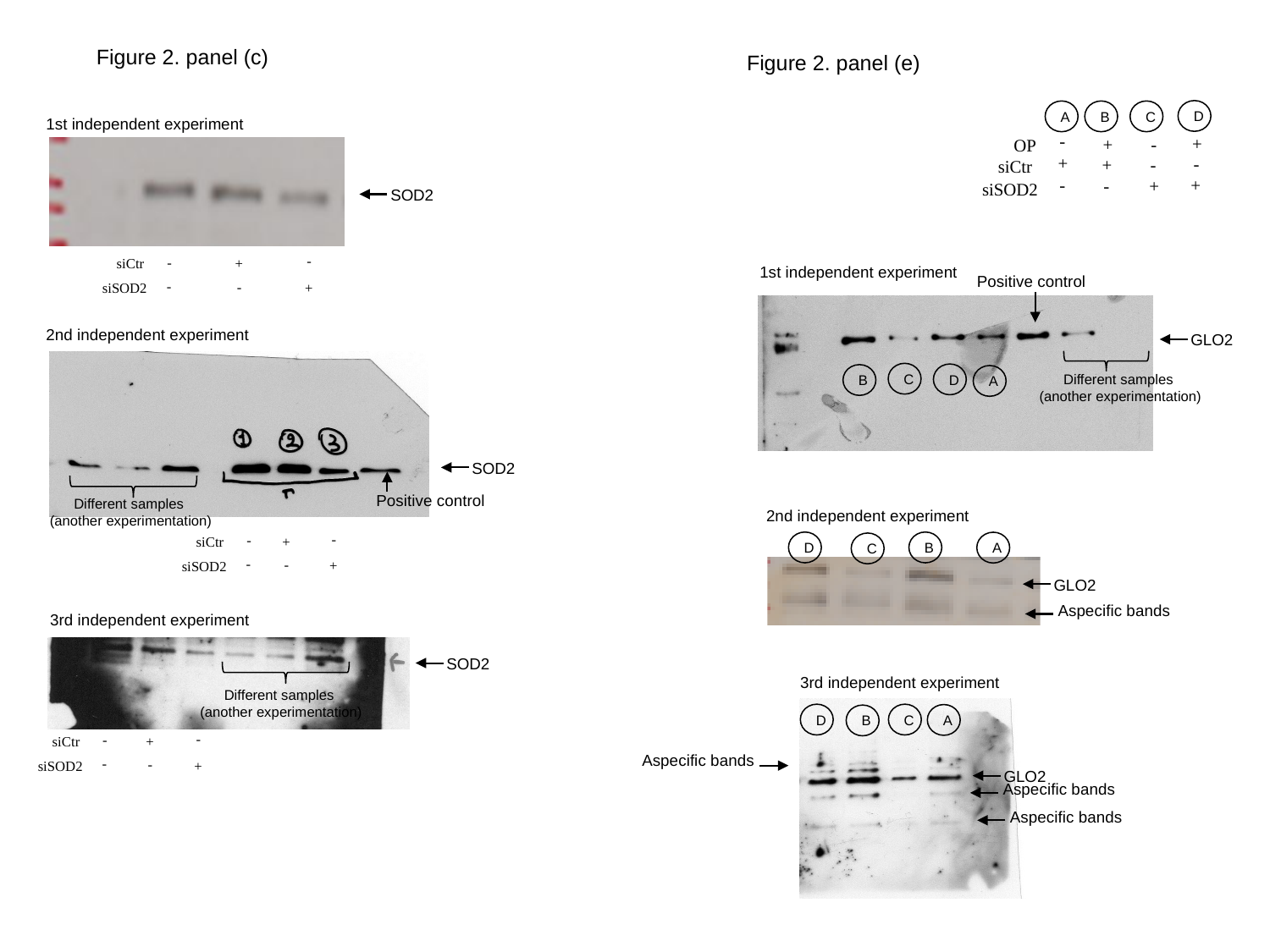

Figure 2. panel (c)
1st independent experiment
SOD2
-
-
siCtr
+
-
-
+
siSOD2
2nd independent experiment
SOD2
Positive control
Different samples
 (another experimentation)
-
-
siCtr
+
-
-
+
siSOD2
3rd independent experiment
SOD2
Different samples
 (another experimentation)
-
-
siCtr
+
-
-
+
siSOD2
Figure 2. panel (e)
D
B
C
A
-
+
+
-
OP
+
-
+
-
siCtr
-
+
-
+
siSOD2
1st independent experiment
Positive control
GLO2
C
D
Different samples
 (another experimentation)
B
A
2nd independent experiment
D
B
A
C
GLO2
Aspecific bands
3rd independent experiment
D
C
A
B
Aspecific bands
GLO2
Aspecific bands
Aspecific bands
