# Supplementary material for: Corrigendum to “Oleuropein-Induced Apoptosis Is Mediated by Mitochondrial Glyoxalase 2 in NSCLC A549 Cells: A Mechanistic Inside and a Possible Novel Nonenzymatic Role for an Ancient Enzyme”
Source: Oxid Med Cell Longev. 2020 Sep 9;2020:3045908. doi: 10.1155/2020/3045908 (PMC7502119; doi:10.1155/2020/3045908)
Supplement: Supplementary Materials — The authors provided a “letter of clarification,” the original files for Figures 2 and 4, and independent replicates. [file 3045908.f1.zip › Independent experiments Fig. 4 (1).pptx]

## Slide 1
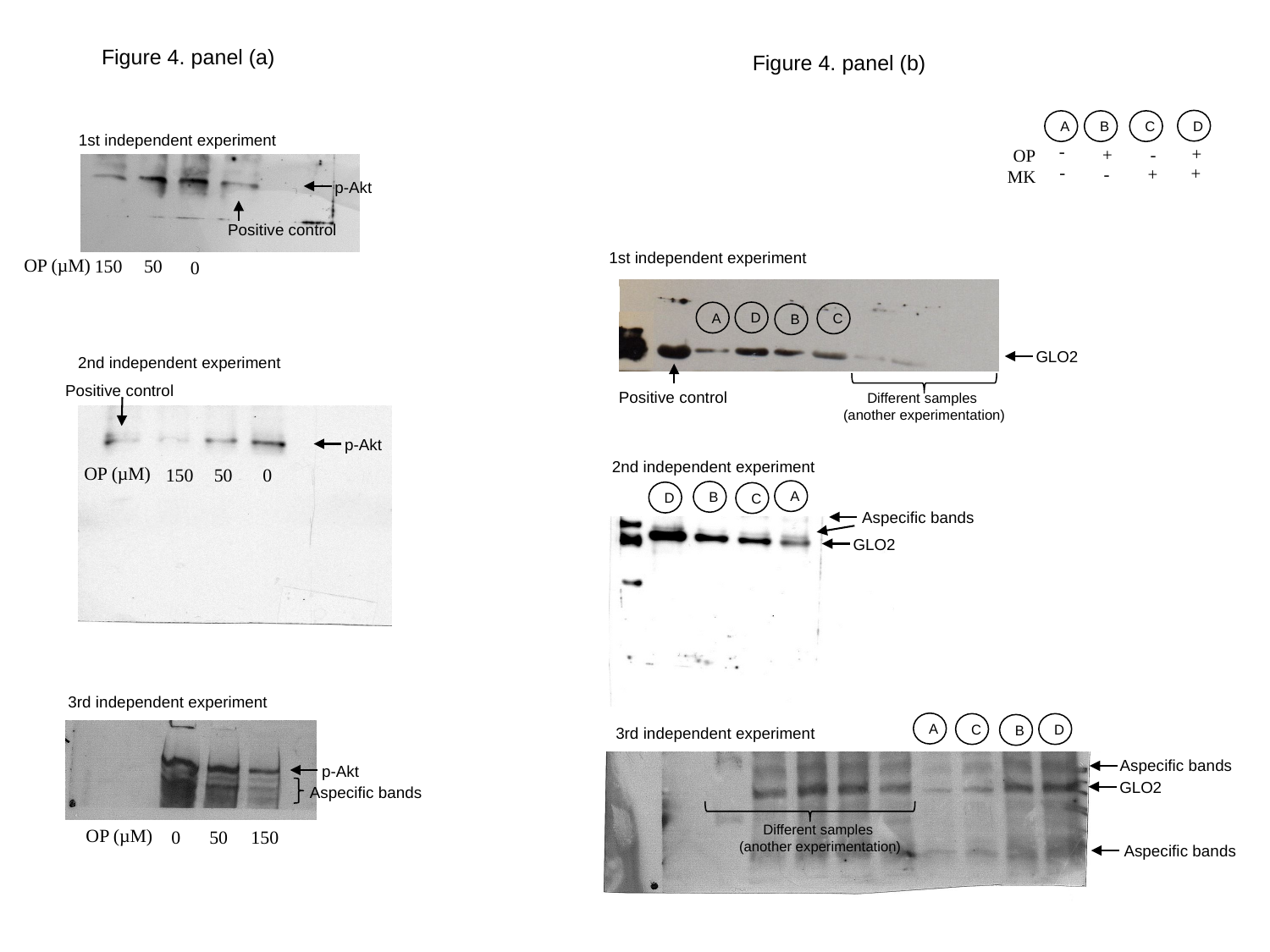

Figure 4. panel (a)
Figure 4. panel (b)
D
B
C
A
-
+
+
-
OP
-
+
-
+
MK
1st independent experiment
p-Akt
Positive control
1st independent experiment
OP (µM)
50
150
0
D
A
C
B
GLO2
Positive control
2nd independent experiment
Positive control
p-Akt
OP (µM)
150
50
0
Different samples
 (another experimentation)
2nd independent experiment
A
B
D
C
Aspecific bands
GLO2
3rd independent experiment
p-Akt
Aspecific bands
OP (µM)
0
50
150
A
C
D
B
Aspecific bands
GLO2
Different samples
 (another experimentation)
Aspecific bands
3rd independent experiment
